# When does the forgetting of trait-implying behaviors affect subsequent person impressions?

**DOI:** 10.3758/s13423-025-02796-1

**Published:** 2026-01-05

**Authors:** Almut Hupbach, Irmak Olcaysoy Okten

**Affiliations:** 1 Department of Psychology, Lehigh University, Bethlehem, PA USA; 2 Department of Psychology, Florida State University, Tallahassee, FL USA

**Keywords:** Directed forgetting, Selective memory, Trait inference, Impression updating, Behavior predictions

## Abstract

Previous research shows that being directed to forget (or remember) trait-implying behaviors immediately after encoding impairs memory for behaviors but not inferred character traits, as measured by the false recognition paradigm. We reassessed this finding using a more diverse set of faces, newly piloted behaviors and traits, and a different trait-inference measure – the savings in relearning paradigm (Experiment 1). After encoding faces with trait-implying behaviors, each followed by remember or forget instructions, participants learned face-trait word pairs in which traits were either consistent or inconsistent with the encoded behavior. Participants recalled more consistent than inconsistent trait words, confirming spontaneous trait inferences during behavior encoding. This effect was resistant to forget instructions, replicating previous findings while addressing limitations of the false recognition paradigm. Experiment 2 replicated impaired recall for forget-cued behaviors using our new materials. Experiment 3 further examined the impact of forget instructions on impression formation and use, specifically whether they influence future behavior predictions. Results showed that directing participants to forget (or remember) trait-implying behaviors reduced expectations of future trait-consistent behaviors and increased openness to trait-inconsistent behaviors. This is the first study to demonstrate that directed forgetting can alter expectations about others, indicating that reduced memory accessibility, whether of impressions or original behaviors, can promote greater flexibility in social judgments. These findings inform theories of directed forgetting and impression formation and have practical implications for contexts where forgetting is both warranted and beneficial.

## Introduction

When people observe others engaging in behaviors suggestive of specific traits, they rapidly and automatically infer and attribute those traits to the individual, a process known as *spontaneous trait inference* (STI; Winter & Uleman, 1984; for a recent review, see Hamilton & Stroessner, 2020; for a meta-analysis, see Bott et al., 2024). Character traits are often inferred based on minimal input and without conscious awareness, even when situational factors or an individual’s goals could plausibly explain the behavior. The implicit nature of STIs makes them difficult to change. For instance, when new trait-inconsistent behavior is introduced or the previous behavior is qualified by additional information, individuals often do not revise their original STIs. Instead, they form new STIs that coexist in memory alongside the original inferences (Olcaysoy Okten et al., 2019; Olcaysoy Okten & Moskowitz, 2020).^1^

Hupbach et al. (2022) asked whether the formation of STIs could be prevented by active forgetting attempts. Participants were informed that they were taking part in a selective memory study and were presented with faces paired with trait-implying behaviors (e.g., “While she was babysitting, she lost track of the 2-year-old”). Following each face-behavior pair, participants were instructed either to remember or to forget that information because it would or would not be tested later. Some of the behavior descriptions contained the implied trait word, but the crucial ones omitted it. Forget instructions impaired participants’ recall of the behaviors but did not reduce false recognition of the inferred (i.e., not presented) traits (e.g., “Irresponsible”). This finding suggests that directed forgetting (DF) primarily impacts memory for specific details (i.e., behaviors) but not for broader gist information (i.e., traits; cf. Ahmad et al., 2019; Fawcett et al., 2016; but see impaired gist or category level memory for semantically related word lists, Marche et al., 2005; Montagliani & Hockley, 2019), and reinforces the notion that traits are automatically inferred at encoding and rapidly integrated into person representation (Todorov & Uleman, 2003). This pattern is also consistent with prior work showing that DF can impair associative memory beyond item-level memory (Hockley et al., 2016; Whitlock et al., 2022), suggesting that forget cues may disrupt associative links between a person and their observed behaviors. The current study aimed to replicate Hupbach et al.’s finding of impaired behavioral recall using a different stimulus set, and to test whether the pattern of robust STIs despite impaired memory for the prompting behaviors would replicate with a different STI measure.

Although often used to measure STIs, the false recognition paradigm has limitations (Orghian et al., 2017), especially when combined with a DF paradigm. Participants who recall behaviors in detail may reject a trait word not because they did not infer it but because they remember it was not explicitly mentioned. Because remember-cued behaviors are better retained, this memory strength difference could mask DF effects on trait inference. In other words, a possible updating in STIs might have remained undetected if the detailed behavior memory in the remember condition reduced the endorsement of inferred traits and thereby eradicated potential differences between the two conditions. On the other hand, previous word-learning studies have shown that remember cues can also increase false alarms due to stronger conceptual activation (Marche et al., 2005; Montagliani & Hockley, 2019; Reid et al., 2023). These mixed findings further complicate the interpretation of recognition-based STI measures and underscore the need for STI paradigms that minimize confounds from memory strength and response biases.

Successfully replicating Hupbach et al.’s (2022) findings with an alternative STI measure would strengthen the conclusion that trait inferences survive active forgetting attempts. Alternatively, showing that forget instructions reduce STIs would be of theoretical and practical importance. Theoretically, it would suggest that active forgetting can prevent the long-term storage of trait information. Practically, this could be relevant in contexts where individuals are exposed to potentially biased or prejudicial information (e.g., jury deliberations or hiring processes; Capers et al., 2018; Wissler et al., 2000). In such settings, individuals may be explicitly instructed by an authority (e.g., a judge) or motivated by institutional policies (e.g., equitable hiring practices) to disregard specific information. By leveraging techniques rooted in active forgetting, it may be possible to promote fairer evaluations by minimizing the impact of irrelevant or outdated trait impressions.

Another goal of the present study was to expand the exploration of DF on person impressions by examining not only whether forgetting influences STIs but also whether it affects how individuals use these impressions when predicting others’ future behaviors. This distinction is important as recent research has revealed dissociations between STI assessments and behavior predictions (Olcaysoy Okten et al., 2019; Olcaysoy Okten & Moskowitz, 2024). Specifically, trait impressions formed from a single behavior tend to persist even after exposure to contradictory information, whereas behavior predictions are more sensitive to new input. Participants continued to recall their initial trait inferences but adjusted their expectations about how the person would act in the future, showing that trait memory and behavioral forecasting are affected differently by new information. Therefore, Experiment 3 tested if behavior predictions are similarly sensitive to DF attempts.

In Experiment 1, we used the savings-in-relearning paradigm (Carlston & Skowronski, 1994) to re-assess the effects of DF on trait inferences. In this paradigm, participants first encode faces paired with trait-implying behaviors. Later, the same faces are presented again paired with trait words. Some of the traits are implied by the previous behaviors, whereas others are new. When participants are asked to recall these trait words using the faces as cues, a benefit is observed for implied over new trait words – a phenomenon known as *savings in relearning*. This suggests that traits were inferred (and thus learned) during the initial encoding of behaviors, facilitating their subsequent (re)learning. While this paradigm also assesses STIs through memory for implied traits, it focuses on trait memory independent of memory for the original behaviors, addressing the confounds associated with the DF procedure in the false recognition paradigm. Moreover, comparing DF effects on behavior recall and trait recognition in the false recognition paradigm is complicated by differences in retrieval demands: retrieval candidates are provided in trait recognition, but must be generated from memory in behavior recall. Asking participants to actively recall trait words in the savings in relearning paradigm better matches the retrieval demands of the behavior recall.

In line with studies using the savings-in-relearning paradigm (Bott et al., 2024), we predict that traits implied by encoded behaviors will be better remembered than traits that contradict these behaviors. Crucially, if STIs are resistant to active forgetting (Hupbach et al., 2022), we expect no difference in the learning of traits associated with forget-cued versus remember-cued behaviors. However, if confounds or enhanced retrieval support have masked DF effects on STIs in the false recognition paradigm, we should instead observe reduced recall of implied traits in the forget condition.

## Experiment 1

### Methods

#### Design and participants

Experiment 1 followed a 2 (cue: forget vs. remember) x 2 (trait match: match vs. mismatch) factorial design with both factors manipulated within subjects. Sample size considerations were based on Hupbach et al. (2022, Exp. 3, *N* = 100) and the meta-analytic finding that effects in the savings-in-relearning paradigm tend to be smaller than those observed in the false recognition paradigm (Bott et al., 2024). To ensure sufficient power to detect an interaction effect between cue and trait match, we initially aimed for a sample size of 120. However, we oversampled to account for anticipated exclusions. We recruited 72 participants from Prolific and 73 participants from the Undergraduate Participant Pool of a university located in Northeast USA. Recruitment on Prolific was limited to native English speakers between the ages of 18 and 35 years residing in the USA, and participants were compensated with $4. University students received research participation credit towards their Introduction to Psychology course grade. As an attention/manipulation check, we asked participants at the end of the experiment whether they had attempted to forget the forget-cued face-behavior pairs. Participants who failed this check or did not recall any trait words were excluded from analyses (*N* = 26). This resulted in a final sample size of 119 participants (66 from Prolific, 53 from the university, 67 women, 51 men, 1 non-binary, age range: 18–36 years, *M* = 23.98, *SD* = 5.85). Information on participants’ race and ethnicity was not collected. Importantly, because the source of participants did not significantly influence trait recall, we collapsed the data across sources.

#### Materials

*Trait word selection.* Behaviors and traits were selected based on a pilot study with 41 undergraduate students (13 women, 28 men; age range = 18–21 years, *M* = 19.1, *SD* = 1.02) from the same university. Participants were presented with 48 trait-implying behaviors: 25% male-negative, 25% male-positive, 25% female-negative, and 25% female-positive. Behaviors included a combination of behaviors drawn directly from previous studies (Ham & Vonk, 2003; Lee et al., 2015, 2017; Olcaysoy Okten & Moskowitz, 2020; Uleman et al., 1996; Winter & Uleman, 1984), modified versions of previously used behaviors updated to better fit today’s context, and newly developed behaviors created specifically for this study. For each behavior, participants were explicitly asked to infer a character trait and type the corresponding trait word into a text box. From the responses, we selected six positive and six negative behaviors for both female and male protagonists, with high agreement on the inferred trait word (.34^2^ to.90), ensuring no repetition of traits across behaviors. For the learning task, the matching trait was always the most frequently inferred trait for a given behavior. For each matching trait, we identified a corresponding mismatching trait for the mismatch control condition through internet searches for antonyms, again ensuring no overlap with other trait words. The full behavior list is available in the Online Supplementary Materials (https://osf.io/z8m9j/?view_only=7eef8a54113a4152882433243ccc61d3).

*Face selection.* Twenty-four images of faces of young individuals with neutral expressions (12 female and 12 male) were selected from the Chicago Face Database (Ma et al., 2015). The set consisted of three faces from each of four distinct racial/ethnic groups (Black, Asian, Latino, and White) for both females and males. Faces were chosen to be visually distinct from one another within each racial/ethnic and gender group to facilitate clear distinction between individuals.

*Face-behavior pairings.* Face-behavior pairings were consistent across participants, with each race-gender combination paired with at least one positive and one negative behavior.

*Assignment of face-behavior pairs to study and test conditions.* Half of the behavior-face pairs in each valence/gender category were followed by a forget cue, and the other half by a remember cue. For the learning task, half of the forget-cued and half of the remember-cued faces were paired with a matching trait, while the other half were paired with a mismatching trait. Four study versions were created by systematically swapping the forget/remember instructions and the matching/mismatching traits.

**Fig. 1:**
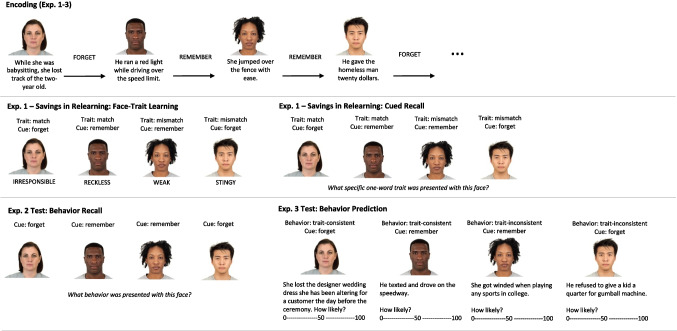
Illustration of encoding and test procedures in Experiments 1–3. The encoding conditions were the same for all experiments. During encoding, half of the face-behavior pairs were followed by a forget and half by a remember cue. In Experiment 1, encoding was followed by face-trait learning with matching and mismatching traits, and the final test was a cued recall of traits. In Experiment 2, cued recall of behaviors was assessed. In Experiment 3, for each face, trait-consistent and trait-inconsistent behaviors were presented, and participants were asked how likely the person is to perform the behavior on a scale from 0 to 100

All experiments were programmed in Qualtrics. Examples for encoding and test materials are presented in Fig. 1.

#### Procedure

The study was introduced as a study on selective memory assessing how well people can remember important information and disregard irrelevant information. Participants were informed that they would be presented with images of faces paired with behavior descriptions, and that some of the face-behavior pairs were important to remember for a later memory test, whereas others were irrelevant, because they would not be tested later. In the study phase, 24 face-behavior pairs were presented one after the other in random order, each for 10 s. Each face-behavior pair was preceded by a 1-s fixation cross. After each presentation, the cue word “remember” or “forget” was displayed for 2 s. After the last face-behavior pair was presented, four unrelated filler questions were asked (e.g., “what did you have for breakfast today”). Then, participants were told that they would see faces again, but this time, each face would be paired with a trait word, and all pairs were important to remember. Face-trait pairs were presented for 10 s each in random order, each preceded by a 1-s fixation cross. After answering a second set of four short filler questions (e.g., “what is your favorite color”), participants were asked to recall the specific trait word that had been presented with each face, by typing it into a text box. If they could not remember the trait word, they were instructed to type “forgot.” The entire procedure took about 20 min.

Two independent raters scored trait recalls. Responses were considered correct if they matched the target trait or contained close synonyms or terms with closely related meanings (e.g., *giving* for generous; *honest* for truthful). Interrater reliability was assessed using Cohen’s kappa (κ =.98, *n* = 1,152), indicating high agreement between raters. Any discrepancies were resolved through discussion.

### Results and discussion

For all studies, we ran mixed-effect models using the *lme4* package in *R* software.

**Fig. 2:**
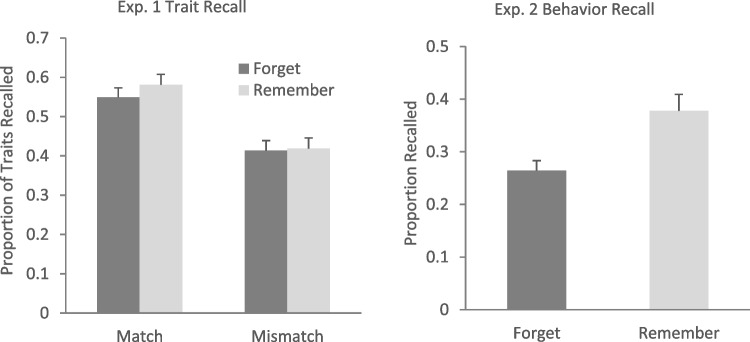
Mean proportion of recognized traits as a function of cue and trait match (Experiment 1, left panel) and proportion of recalled behaviors as a function of cue (Experiment 2, right panel). Error bars represent standard errors of the mean

Trait recall was estimated as a categorical dependent variable using the *glmer* function, with cue (forget vs. remember) and trait match (match vs. mismatch) as predictors. The best model fit was observed when the random intercept for traits and subjects along with random slopes for the effect of trait match across subjects and for the effect of trait match across traits were included. The model’s estimates are provided in Table 1, and the mean proportion of recalled traits is plotted in Fig. 2 (left panel). A significant fixed effect of trait match was observed; participants recalled more traits that matched the behavior than traits that mismatched it, consistent with a savings (i.e., STI) effect.^3^ Importantly, neither the fixed effect of cue nor the interaction between cue and trait match were significant, suggesting that savings were not affected by the instruction to remember or forget. We supplemented this analysis with Bayesian logistic mixed-effects models using the *stan_glmer()* function from the *rstanarm* package. To evaluate whether the interaction between cue type and match condition improved model fit (as an indicator of a DF effect on STIs), we compared a full model (including the interaction) to a reduced model (excluding the interaction) using Bayes factors estimated via bridge sampling. The Bayes factor (BF₁₀ = 0.01) generated from this comparison indicated that the model with the interaction was substantially less likely given the data than the model without it. This provides strong evidence that the interaction did not meaningfully improve explanatory power, suggesting the absence of a DF effect on STIs. Full model details, including coefficient estimates, credible intervals, and diagnostic checks, are reported in the Online Supplementary Materials.

**Table 1:** Main effects and interactions from the mixed-effects binomial regression model on trait recall (Model <- *glmer*(traitrecall ~ cue + traitmatch + cue * traitmatch + (1+traitmatch|ID) + (1+traitmatch|trait), data =., family = binomial, control = glmerControl(optimizer = "bobyqa"))

	Estimate	*SE*	*z*	*p*
Intercept	−0.04	0.16	−0.26	.79
Cue	0.11	0.09	1.30	.20
Trait Match	0.79	0.14	5.72	<.001
Cue x Trait Match	0.12	0.17	0.67	.50

Together, results show savings in learning of traits that were consistent with the encoded behaviors, suggesting that participants inferred the traits during the encoding (Carlston & Skowronski, 1994). Importantly, savings were unaffected by the forget cue, supporting Hupbach et al.’s (2022) finding that trait inferences are resistant to active forgetting and underscoring the robustness of this phenomenon across measures. However, because we used somewhat different materials to Hupbach et al. (a diverse representation of ethnicities and races to ensure greater inclusivity and more accurate representation of the US population and some novel behaviors), it is important to confirm that forget instructions still impair behavior recall. We conducted a second experiment with the same face-behavior pairs, predicting that we would replicate Hupbach et al.’s finding of reduced recall for forget-cued versus remember-cued behaviors.

## Experiment 2

### Methods

The materials (face-behavior pairs) and initial encoding procedure were the same as in Experiment 1. We omitted the face-trait learning and changed the memory test. Specifically, after participants encoded the face-behavior pairs (and a short distraction phase), we presented the faces again in a random order and asked participants to report the associated behavior by typing it into a text box. We explicitly told participants to recall behaviors even if they had been instructed to forget them. If they could not remember the behavior, they were instructed to type “no memory.” The entire procedure took about 20 min. To analyze behavior recalls, each behavior was divided into four content units (e.g., She waited/for her dinner order/for 90 minutes/without complaining). For each recalled unit, 0.25 points were awarded, with a maximum of 1 point for recalling all units of a behavior. Three independent raters evaluated the recalls, resolving discrepancies by majority vote or, if all three scores differed, through discussion. Interrater reliability was assessed using the intraclass correlation coefficient (ICC), based on a two-way mixed-effects model for consistency and the reliability of a single rater’s score (ICC[3,1]; Shrout & Fleiss, 1979). The resulting ICC was 0.974, suggesting that the raters applied the scoring criteria with a high degree of consistency.

The experiment followed a within-subjects design with one factor: behaviors were followed by either a forget or a remember cue. Based on Hupbach et al. (2022, Exps. 1a and 1b), we aimed for a sample size of 50 participants, and recruited 59 participants (US residents, fluent in English) from Prolific. We excluded participants who recalled fewer than 5% of behaviors (Hupbach et al., 2022) or mentioned in the attention/manipulation check that they did not attempt to forget the forget-cued behaviors. The final sample consisted of 49 participants (27 women, 21 men, one non-binary) between the ages of 19 and 40 years (*M* = 27.51, *SD* = 5.01 years). Information on participants’ race and ethnicity was not collected. Participants received $4 for their participation.

### Results and discussion

Figure 2 (right panel) shows the mean proportion of recalled behaviors. Recall was estimated through the *lmer* function. The best model fit was observed when the random intercepts for behaviors and subjects as well as a random slope for cue across subjects were included. As expected, cue (forget vs. remember) affected behavior recall, *t*(47.27) = 4.05, *p* <.001, showing that fewer behaviors were recalled when participants had been instructed to forget the face-behavior pairs.

Experiments 1 and 2 indicate that DF induces forgetting of specific behaviors, while inferred traits continue to shape subsequent trait learning. Consistent with Hupbach et al. (2022), these results suggest that trait inferences are resistant to active forgetting. However, while memory-based STI assessments reliably capture STIs (Bott et al., 2024), they might lack the sensitivity needed to detect more subtle shifts in impressions. Indeed, recent work showed that when participants were asked to predict others’ future behaviors, they utilized additional contradictory behavioral information that was presented as part of a second learning phase, despite continued endorsement of the originally inferred trait from the first learning phase in a false recognition test (Olcaysoy Okten & Moskowitz, 2024). This dissociation suggests that focusing on a single trait may obscure the complexity of an individual’s overall impression. Behavior predictions may promote more reflective evaluation processes, enabling a more nuanced assessment of impressions. Importantly, behavior predictions also reveal how people integrate STIs into broader evaluative processes and decision-making about others (McCarthy & Skowronski, 2011).

Would active forgetting of behaviors make people more cautious in predicting future behaviors? In Experiment 3, after having encoded faces paired with trait-implying behaviors followed by either forget or remember instructions, we asked participants to predict the likelihood of protagonists’ trait-consistent and trait-inconsistent behaviors. We predicted that forget cues would not influence these predictions, because behavior predictions are independent of the perceiver’s ability to recall the original trait-implying behaviors (McCarthy & Skowronski, 2011), and because of the assumption that traits are automatically inferred and instantaneously stored in memory. However, asking about the likelihood of specific future behaviors might encourage more reflective processes that could lead to a more nuanced evaluation of the person than what is assessed by asking about memory for specific traits. If so, we might see DF effects in behavior predictions in the form of a weaker endorsement of trait-consistent behaviors of actors whose behaviors were cued to be forgotten (vs. remembered) or perhaps even in the form of a stronger endorsement of trait-inconsistent future behaviors.

## Experiment 3

The methods, predictions, and analytic approach of Experiment 3 were preregistered on the Open Science Framework (https://osf.io/eu8tk/?view_only=fe51ee1e60df4eb3af0b157ca6f4de66).

### Methods

#### Design and participants

The experiment followed a 2 x 2 factorial design, with both factors manipulated within subjects: behaviors were followed by either a forget or a remember cue during encoding, and faces were paired with trait-consistent and inconsistent behaviors during test. We recruited 100 participants (US residents, fluent in English) from Prolific. As preregistered, we excluded two participants who responded with the same rating (0, 50) during test for more than 80% of the trials. The final sample consisted of 98 participants (58 women, 34 men, six non-binary/non-conforming) between the ages of 18 and 45 years (*M* = 29.53, *SD* = 7.69 years). Fifty participants identified as White (51%), 26 as Black or African American (27%), 12 as Asian (12%), four as Multiracial (4%), two as American Indian or Alaska Native (2%), and four preferred not to answer. Participants received $4 for their participation.

#### Materials

The same 24 face images and behaviors as in Experiments 1 and 2 were used in Experiment 3. Unlike in previous experiments, we not only counterbalanced the forget and remember cues across participants but also varied the assignment of the protagonist’s gender to the behaviors, resulting in four study versions. Specifically, behaviors followed by a forget cue in one version were followed by a remember cue in another. Additionally, within each of these two versions, we created two subversions by varying the gender of the protagonist performing the behavior. Each participant viewed an equal number of male and female faces.

Trait-consistent and -inconsistent behaviors for the behavior prediction test phase were selected based on pilot studies. Specifically, we recruited student participants from a university located in the Southeast USA for four rounds of piloting (*N* = 47, *N* = 47, *N* = 48, *N* = 95). These pilot studies included a collection of new behaviors that were created to imply the same (original) or the opposite traits to the ones implied in our original set of behaviors used in Experiments 1 and 2. For example, if the original behavior implied the trait “irresponsible,” a consistent behavior was created to imply “irresponsible,” and an inconsistent behavior was created to imply “responsible.” Participants rated the degree to which each version of the behaviors (the original, trait-consistent, and trait-inconsistent) performed by actors with gender-neutral names implied the original trait (e.g., “To what extent do you think the trait “irresponsible” represents this person’s trait?; 1: *Not at all*, 7: *Very well*). Selected trait-consistent behaviors implied the given trait to the same degree as the original behavior (*p*s >.09, *d*s <.25) and trait-inconsistent behaviors implied those traits to a significantly lower degree (*p*s <.001). We provide the details of those pilot studies in the Online Supplementary Materials (see Table S2 for the final list; https://osf.io/z8m9j/?view_only=7eef8a54113a4152882433243ccc61d3).

#### Procedure

The encoding procedure was identical to that used in Experiments 1 and 2. After all 24 face-behavior pairs were presented and the same short filler task as in Experiment 2, participants were informed that they would see the same faces again. This time, as part of the behavior-prediction task, they were instructed to rate how likely the depicted person was to engage in a specific behavior (different from the one they had previously seen), using a scale from 0 to 100 in increments of 10. Participants were informed that there were no right or wrong answers and that they should follow their first impression. Each face was presented twice, once with a trait-consistent and once with a trait-inconsistent behavior. Test pairs were presented in a randomized order.

### Results and discussion

**Fig. 3:**
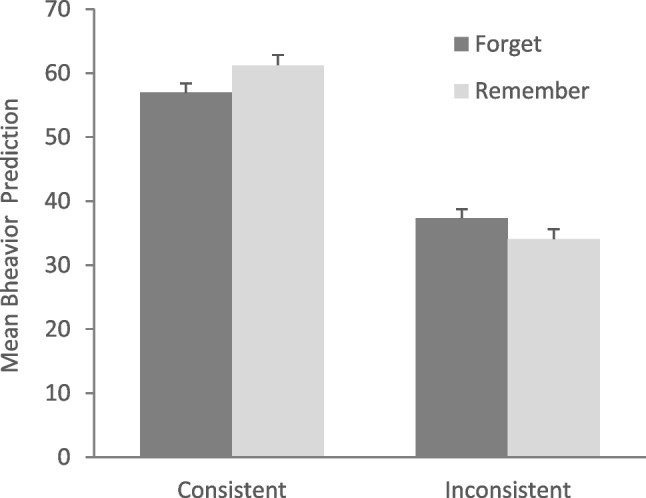
Mean likelihood of behavior (0–100) as a function of cue and trait consistency of behavior (consistent vs. inconsistent). Error bars represent standard errors of the mean

Behavior predictions were estimated through the *lmer* function. The best model fit was achieved when random intercepts for behaviors and subjects were included, along with random slope for the effect of behavior consistency across subjects. Table 2 shows the estimated fixed effects, and Fig. 3 shows the mean endorsements of future behaviors. As predicted and preregistered, we found a significant effect of consistency, with trait-consistent behaviors being endorsed more strongly than trait-inconsistent behaviors. We did not find a main effect of cue but unexpectedly found a cue by consistency interaction. Post hoc comparisons using the *emmeans* package in R revealed significantly higher endorsements of trait-consistent behaviors in the remember in comparison to the forget group (*p* <.001), but the opposite for trait-inconsistent behaviors, i.e., significantly higher endorsements of trait-inconsistent behaviors in the forget in comparison to the remember group (*p* =.003).

**Table 2:** Main effects and interactions from the mixed-effects linear regression model on behavior predictions (Model <- *lmer*(prediction ~ cue + consistency + cue * consistency + (1+consistency|ID) + (1 |behavior))

	Estimate	*SE*	*df*	*t*	*p*
Intercept	47.34	0.90	73.83	52.56	<.001
Cue	0.27	0.40	4508.52	0.68	.50
Consistency	11.69	1.15	97.01	10.18	<.001
Cue x Consistency	1.90	0.39	4482.45	4.82	<.001

These findings suggest that forget instructions weaken the associations between person and traits in memory, such that trait-consistent future behaviors are predicted with less certainty, and trait-inconsistent behaviors are evaluated as more likely. This provides initial evidence that DF can modulate how perceivers apply trait information when forming expectations about others’ future behavior. This in turn qualifies our previous conclusion that first impressions are immune to DF instructions. Instead, a more nuanced picture emerges where forgetting instructions do not “erase” trait associations but diminish their influence on subsequent person evaluations. Traits associated with forget-cued behaviors can be accessed when directly queried, as demonstrated by false recognition of these traits (Hupbach et al., 2022) and by savings in learning behavior-consistent traits (Experiment 1). However, the role these traits play in predicting behaviors is reduced via DF.

## General discussion

The present work documents two important findings regarding impression and memory updating. First, it replicated a recently identified phenomenon, the directed forgetting (DF) of others’ behaviors yet not the spontaneously inferred traits (STIs) from those behaviors, with a paradigm that addresses the limitations of the previously used false recognition paradigm in that research. Specifically, the savings in relearning paradigm provided a methodological advantage in terms of minimizing the effect of behavior memory on the responses for trait memory while also equalizing the task demands across the trait memory task used in Experiment 1 and the behavior memory task used in Experiment 2 (both tasks were recall tasks). Second, it demonstrated that although STIs were robust to DF instructions, perceivers’ predictions about actors’ future behaviors were nonetheless influenced by those instructions. Specifically, Experiment 3 showed that after being cued to forget (vs. remember) an actor’s initial behavior, participants made weaker predictions about trait-consistent behaviors and stronger predictions about trait-inconsistent behaviors – indicating impression updating. We interpret these behavior predictions as reflecting perceivers’ overall impressions of the actors. While STI measures such as the false recognition paradigm and the savings-in-relearning paradigm capture whether trait information is spontaneously inferred, behavior predictions provide a measure of how such information is applied when reasoning about others in new contexts. In this way, our findings suggest that DF does not necessarily eliminate trait inferences but weakens their influence on subsequent social judgments. Further, the unexpected finding of strengthened predictions for trait-inconsistent behaviors may suggest that DF helps to negate consistent *and* affirming inconsistent judgments simultaneously, though more research is needed to unravel the mechanism of this unexpected finding (see Mayo et al., 2004).

The present work also offers broader implications for impression and memory research. It adds to the growing body of evidence indicating that memory-based measures of impressions can diverge from prediction-based measures (Olcaysoy Okten et al., 2019; Olcaysoy Okten & Moskowitz, 2024). Specifically, our results indicate that people are more cautious when endorsing future trait-consistent behaviors, particularly when given time and opportunity to reflect, and more willing to entertain the possibility of trait-inconsistent behaviors. When asked to assess how someone will behave in the future, perceivers may not exclusively rely on the inferred trait or gist information but may also consult their memory for previous behaviors. Our findings support this possibility as forget-cued behaviors are more frequently forgotten than remember-cued behaviors (Experiment 2), and so are impressions measured via a prediction-based measure (Experiment 3). One limitation of the study is that it did not directly assess the relationship between behavior recall and behavior predictions, leaving open the question of how participants approached the prediction task. Clarifying the mechanisms underlying behavior predictions could inform theories of DF and impression formation and should be undertaken in future research. If attempts to forget weaken actor-trait associations, this would challenge the assumption that DF affects only verbatim memory while leaving gist representations intact (Ahmad et al., 2019; Fawcett et al., 2016; Hupbach et al., 2022) and would align more with word-list studies showing impaired verbatim *and* gist memory (Marche et al., 2005; Montagliani & Hockley, 2019). It would further inform where in the three-step sequence of trait inference (Carlston & Skowronski, 1994; Mae et al., 1999) interventions could make a difference. While forget cues presented after trait-implying behaviors are unlikely to disrupt the initial trait-activation phase, they could plausibly influence either the strength of association between actors and traits (i.e., the association phase) or the long-term binding of traits to actors. Understanding these dynamics could provide deeper insights into how memory processes shape social impressions over time.

The current study focused primarily on possible DF effects on trait-based impressions, as traits typically operate as effective cognitive organizers (i.e., the gist) in impression formation (Hamilton et al., 1989). However, other types of associations relevant to impression formation such as affective associations (Amodio & Ratner, 2011) may also be susceptible to DF effects. Using a list-wise DF paradigm, Scully and Hupbach (2020) found that instructing participants to forget (vs. remember) negative and neutral behaviors of a fictional actor not only reduced memory for those behaviors but also shifted evaluations of the actor’s warmth and dominance. Specifically, forgetting negative behaviors led to increased ratings of warmth and decreased ratings of dominance, with ratings of warmth negatively correlated and ratings of dominance positively correlated with memory for negative behaviors. These findings highlight the potential for DF to reshape social perceptions by weakening not just cognitive associations but also affective evaluations. Future research should examine the broader impact of item-wise DF of trait-implying behaviors for affective associations. Based on our findings – where participants in the forget condition showed reduced endorsement of trait-consistent behaviors and increased endorsement of trait-inconsistent behaviors – we anticipate similar DF effects on affective associations, potentially altering emotional evaluations alongside cognitive impressions.

Taken together, our study is the first to demonstrate that DF of trait-implying behaviors limits the influence of past behaviors and inferred traits on person impressions and future behavior predictions. This suggests that when people intentionally forget certain behaviors, it makes their impressions of others more flexible and less anchored to their past. These findings have practical implications for situations where forgetting is not only warranted but also beneficial. For instance, in contexts like rehabilitation, forgetting minor past infractions can lead to fairer judgments about a person’s potential for change. In personal relationships, selectively forgetting past conflicts can make room for forgiveness and healthier interactions (Sell, 2016). Understanding how directed forgetting works in these social contexts can help find ways to reduce bias, improve decision making, and encourage more balanced judgments.

## Footnotes

1 In contrast to STIs, implicit attitudes can update dynamically when people encounter highly diagnostic, strongly valenced new information that contradicts the original impression (e.g., Cone et al., 2021; Mann & Ferguson, 2015, 2020).

2 This number represents the most conservative lower boundary, that is, agreement on the exact trait word. Including synonyms or closely related trait words would result in higher numbers.

3 Although we interpret this as a savings effect consistent with STIs, we acknowledge that without a neutral control condition, we cannot determine whether the observed difference reflects facilitation for matching traits, interference for mismatching traits, or both. Prior research including neutral baselines has shown evidence of both processes (Carlston & Skowronski, 1994).

## Declarations

### Open practices statement

Experiment 3 was preregistered (https://osf.io/eu8tk/?view_only=fe51ee1e60df4eb3af0b157ca6f4de66).

Supplementary Materials and data for all experiments are available at: https://osf.io/z8m9j/?view_only=7eef8a54113a4152882433243ccc61d3
